# The Influence of Surgical Weight Reduction on Left Atrial Strain

**DOI:** 10.1007/s11695-021-05710-5

**Published:** 2021-09-22

**Authors:** Jakub Strzelczyk, Piotr Kalinowski, Krzysztof Zieniewicz, Cezary Szmigielski, Michał Byra, Grzegorz Styczyński

**Affiliations:** 1 Department of Internal Medicine, Hypertension and Angiology, Medical University of Warsaw, Stefana Banacha Street 1A, 02-097 Warsaw, Poland; 2 Department of General, Transplant and Liver Surgery, Medical University of Warsaw, Stefana Banacha Street 1A, 02-097 Warsaw, Poland; 3 Department of Ultrasound, Institute of Fundamental Technological Research, Polish Academy of Sciences, Adolfa Pawińskiego Street 5B, 02-106, Warsaw, Poland

**Keywords:** Left atrial strain, Bariatric surgery, Atrial fibrillation, Heart failure

## Abstract

**Background:**

Obesity increases and surgical weight reduction decreases the risk of atrial fibrillation (AF) and heart failure (HF). We hypothesized that surgically induced weight loss may favorably affect left atrial (LA) mechanical function measured by longitudinal strain, which has recently emerged as an independent imaging biomarker of increased AF and HF risk.

**Methods:**

We retrospectively evaluated echocardiograms performed before and 12.2 ± 2.2 months after bariatric surgery in 65 patients with severe obesity (mean age 39 [36; 47] years, 72% of females) with no known cardiac disease or arrhythmia. The LA mechanical function was measured by the longitudinal strain using the semi-automatic speckle tracking method.

**Results:**

After surgery, body mass index decreased from 43.72 ± 4.34 to 30.04 ± 4.33 kg/m^2^. We observed a significant improvement in all components of the LA strain. LA reservoir strain (LASR) and LA conduit strain (LASCD) significantly increased (35.7% vs 38.95%, *p* = 0.0005 and − 19.6% vs − 24.4%, *p* < 0.0001) and LA contraction strain (LASCT) significantly decreased (− 16% vs − 14%, *p* = 0.0075). There was a significant correlation between an increase in LASR and LASCD and the improvement in parameters of left ventricular diastolic and longitudinal systolic function (increase in *E*’ and MAPSE). Another significant correlation was identified between the decrease in LASCT and an improvement in LA function (decrease in *A*’).

**Conclusions:**

The left atrial mechanical function improves after bariatric surgery. It is partially explained by the beneficial effect of weight reduction on the left ventricular diastolic and longitudinal systolic function. This effect may contribute to decreased risk of AF and HF after bariatric surgery.

**Graphical abstract:**

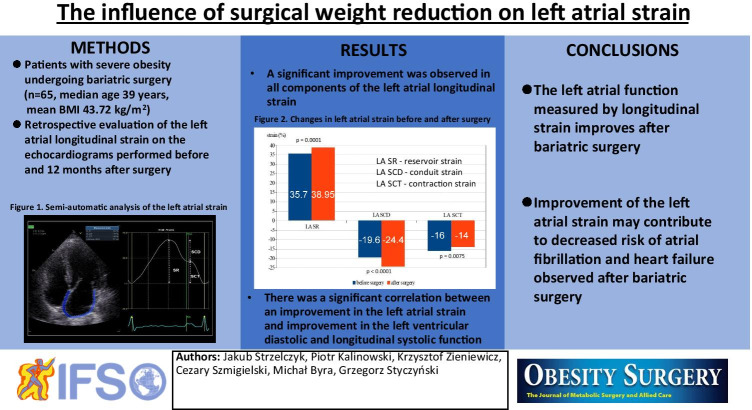

## Introduction

Obesity is known to be associated with many adverse cardiovascular effects, including an increased risk of atrial arrhythmias, mainly atrial fibrillation (AF), and heart failure with preserved ejection fraction (HFpEF) [1, 2]. It is believed that structural and/or functional abnormalities of the left atrium are at the core of the atrial arrhythmia generation [3]. Moreover, left atrial (LA) mechanical dysfunction is known to contribute to the worsening of the left ventricular (LV) filling and to the development of HFpEF [4, 5].

Both increased weight, and obesity-related comorbidities, such as arterial hypertension, may negatively affect LA structure and function, favoring the development of atrial arrhythmia and diastolic dysfunction [6–8].

Traditionally, the LA evaluation in the imaging studies was based first on the assessment of the LA antero-posterior diameter and later on the LA area and on the LA volume. Only recently, the LA longitudinal strain (change in length of the atrial walls during the cardiac cycle) emerged as a promising measure of the LA mechanical function [9, 10]. Several studies suggested independent and superior to other morphological LA measures predictive role of the LA strain for the occurrence of paroxysmal AF in patients with ischemic stroke, hypertrophic cardiomyopathy, chemotherapy, HFpEF, and after coronary artery bypass grafting [11–15]. Moreover, LA strain was shown to independently predict incident HFpEF and to improve diastolic dysfunction classification [16–19]. Data from the literature suggests that the risk of atrial arrhythmia and HFpEF is increased in obesity and with the presence of LA mechanical dysfunction measured by strain. On the other hand, the increased risk of AF and HFpEF in obesity is effectively reduced by surgically induced weight loss [20–24].

Therefore, we hypothesized that weight reduction after bariatric surgery may have a beneficial effect on the LA mechanical function measured by LA strain.

## Materials and Methods

### Patient Population and Study Design

We retrospectively evaluated echocardiograms of patients, who had follow-up echocardiographic examination after 12.2 ± 2.2 months following bariatric surgery (sleeve gastrectomy). The initial cohort consisted of 195 consecutive bariatric patients who had preoperative echocardiographic study ordered by a surgeon to improve perioperative risk assessment. After the exclusion of 24 patients (7, due to significant cardiac disease; 2, due to alcohol overuse; and 15, due to poor cardiac visualization or missing data), 171 subjects were further analyzed. The follow-up study was planned in all patients as a further insight into potentially beneficial cardiovascular effects of the bariatric surgery, however; only 93 patients attended the follow-up examination. Of them, 28 patients had an inadequate quality of the preoperative LA image for performing the LA strain analysis, and therefore, 65 patients were finally included in our study. The clinical characteristic of the included subjects is presented in Table 1.

**Table.1:** Clinical characteristics of patients (*n* = 65)

Age (years)	39 [36; 47]
Female sex, *n* (%)	47 (72)
Height (cm)	169.86 ± 9.35
Body weight (kg)	123 [114; 139]
Body mass index (kg/m^2^)	43.72 ± 4.34
Body surface area (m^2^)	2.41 [2.25; 2.58]
Systolic blood pressure (mmHg)	133 [125; 142]
Diastolic blood pressure (mmHg)	81 [78; 86]
Heart rate (beats per minute)	73 ± 9
Hypertension, *n* (%)	39 (60)
Diabetes mellitus, *n* (%)	17 (26)
Hyperlipidemia, *n* (%)	25 (38)
Active smoking, *n* (%)	16 (25)

Surgery candidates were assessed for historical and active tobacco use during the initial evaluation. All active tobacco users were routinely advised to stop smoking at least 6 to 8 weeks before surgery. However, active smoking was not implemented as an absolute contraindication for surgery and smokers were not disqualified from surgery and smoking reduction declared by patients was acceptable during the study.

### Echocardiographic Evaluation

**Fig. 1:**
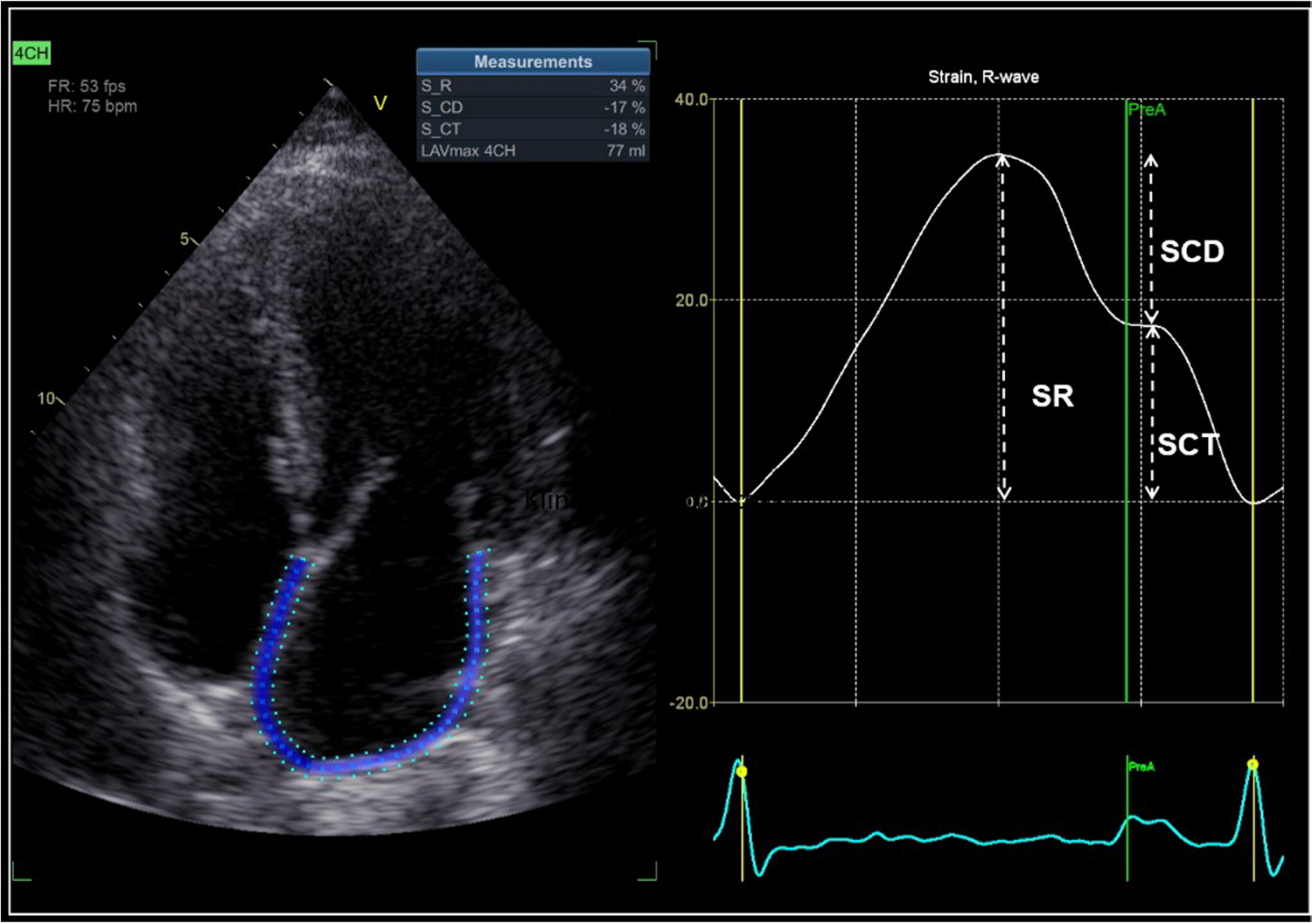
Semi-automatic analysis of left atrial (LA) strain. The solid line on the graph represents changes in the LA strain during the cardiac cycle. The dotted white arrows represent three components of the LA strain: SR, reservoir strain (left atrial elongation during left ventricular systole); SCD, conduit strain (left atrial shortening during early left ventricular diastole); SCT, contraction strain (left atrial shortening during atrial contraction)

Echocardiographic images were acquired using GE Vivid E9 cardiac ultrasound system with M5S-D (1.7/3.3 MHz) probe (GE Healthcare, Horten, Norway) and stored on the Echopac workstation, version 103 (GE Healthcare, Horten, Norway). Left atrial longitudinal strain analysis was performed with semi-automatic speckle tracking method in apical four-chamber view using Echopac 204 version (Fig. 1). Left atrial wall delineation was generated automatically with the option of manual correction, and tracking was visually compared with the underlying motion of atrial walls to ensure optimal quality of tracking. Measurements were performed during one cardiac cycle beginning at the end of diastole represented by an *R* wave on the electrocardiographic tracing.

Left longitudinal strain analysis included assessment of the LA reservoir strain (LASR), which represents elongation of the left atrial walls during ventricular systole and is presented as a positive value. Other parameters, like the LA conduit strain (LASCT), represent shortening of the left atrium during early diastole and therefore is measured as a negative value. LA contraction strain (LASCT) represents further shortening of the left atrium during atrial contraction and also is shown as a negative value. LV end-diastolic dimension, LV wall thickness, aortic root dimension, and LA antero-posterior dimension were all measured in parasternal long-axis views; LA area was measured in apical four-chamber view and LA volume in apical four-chamber and two-chamber views. Due to poor visualization of the endocardial borders in the apical views in a significant proportion of the patients, the LV ejection fraction calculation by the Simpson method and the longitudinal strain assessment were not included in the study measurements. Therefore, other echocardiographic methods for the assessment of the LV systolic function, which are less dependent on the endocardial border delineation, were implemented. Fractional shortening (FS) was used for transverse systolic function assessment. The longitudinal systolic LV function was assessed by the maximal systolic velocity of the mitral annulus by tissue Doppler imaging (LV S) and by the mitral annular plane systolic excursion (MAPSE) by M-mode from the apical four-chamber view. The mean value of the maximal systolic velocity of the lateral and medial part of the mitral annulus and the mean value of MAPSE from the lateral and medial part of the mitral annulus were calculated. LV diastolic function was assessed with the ratio of the early-to-late pulse wave Doppler velocities of the mitral inflow (E/A), the mean of tissue Doppler early diastolic lateral and medial mitral annulus velocities (*E*’), and *E*/*E*’ ratio. The results of the Doppler recordings were averaged from 5 consecutive cardiac cycles. Blood pressure was measured at the end of the echocardiographic examination, using an automated oscillometric monitor (Microlife, Watch BP Office, Switzerland).

### Statistical Analysis

The study design was a retrospective observational analysis. The *W* Shapiro–Wilk test was used to assess the normality of the distribution of variables. For continuous variables with normal distribution, data were expressed as mean and standard deviation. For continuous variables with non-normal distributions, data were summarized as median and interquartile range. Categorical data were presented as a number of cases in each category and percentages. Subsequently, an analysis of the impact of weight loss on the echocardiographic parameters was performed. For the comparison of repeated measurements, Student’s *t* test and the Wilcoxon matched-pairs test were applied for variables with normal and non-normal distribution respectively. Associations between changes in strain values and changes in anthropometric and selected cardiac functional and morphological variables after weight loss were measured using Spearman correlation. *P* values < 0.05 were considered statistically significant. Intra- and interobserver repeatability was tested on the preoperative images of 15 randomly selected patients. Repeatability was expressed as intraclass correlation coefficient (ICC) and as an absolute difference between 2 measurements/means of the 2 measurements, shown in percentages, mean, and standard deviation. All computations were performed using STATISTICA 12.5 (StatSoft, Tulsa, USA).

## Results

In the studied group of patients, the median reduction of body weight 12 months after bariatric surgery was 37 [31; 47] kg, which corresponded to 31.28 ± 7.14% of the initial weight. Mean body mass index (BMI) decreased from 43.72 ± 4.34 to 30.04 ± 4.33 kg/m^2^, *p* < 0.0001. The mean reduction of the basic hemodynamic parameters was for systolic blood pressure (SBP) 9 ± 14 mmHg, *p* < 0.0001, for diastolic blood pressure (DBP) 6 [0; 10] mmHg, *p* < 0.0001, and for heart rate 9 ± 9 beats per minute, *p* < 0.0001. Echocardiographic parameters before and after weight reduction are presented in Table 2.

**Table.2:** Echocardiographic parameters before and after bariatric surgery (*n* = 65)

Parameter	Before surgery	After surgery	*P* value
LV (cm)	4.83 ± 0.36	4.73 ± 0.39	0.005*
LV/BSA (cm/m^2^)	1.99 ± 0.18	2.36 ± 0.25	< 0.0001*
LVM (g)	198.57 [178.22; 230.58]	173.85 [155.55; 200.6]	< 0.0001*
LVMI (g/m^2^)	83 [79; 91]	88 [80; 97]	0.025*
RWT	0.46 ± 0.05	0.43 ± 0.07	0.001*
FS (%)	39.38 ± 6.22	41.45 ± 4.97	0.026*
LV S (m/s)	0.08 ± 0.01	0.09 ± 0.01	0.004*
MAPSE mean (cm)	1.41 ± 0.19	1.46 ± 0.17	0.027*
LA (cm)	4.14 ± 0.28	3.94 ± 0.31	< 0.0001*
LA/BSA (cm/m^2^)	1.69 [1.6; 1.8]	1.95 [1.84; 2.09]	< 0.0001*
LA area (cm^2^)	19.91 ± 2.75	18.05 ± 2.88	< 0.0001*
LA area/BSA (cm^2^/m^2^)	1.69 ± 0.25	1.96 ± 0.18	< 0.0001*
LA volume (ml)	57.68 ± 13.44	51.85 ± 11.56	< 0.0001*
LAVI (ml/m^2^)	23.71 ± 4.83	25.95 ± 5.39	< 0.0001*
LASR (%)	35.70 ± 7.04	38.95 ± 5.97	0.0005*
LASCD (%)	− 19.6 ± 4.96	− 24.4 ± 5.55	< 0.0001*
LASCT (%)	− 16 [− 20; − 12]	− 14 [− 17; − 12]	0.0075*
Ao (cm)	3.28 ± 0.3	3.26 ± 0.33	0.2
*E* (m/s)	0.80 ± 0.15	0.84 ± 0.12	0.035*
*A* (m/s)	0.7 ± 0.12	0.68 ± 0,15	0.162
*E*/*A*	1.14 [0.95; 1.35]	1.25 [1.05; 1.47]	< 0.0001*
*E*’ (m/s)	0.1 ± 0.02	0.12 ± 0.02	< 0.0001*
*A*’ (m/s)	0.1 ± 0.02	0.09 ± 0.02	0.0008*
*E*/*E*’	8.1 [6.93; 8.74]	6.93 [6.23; 8.13]	< 0.0001*

^*^p < 0.05

Abbreviations: *LV*, left ventricular dimension; *BSA*, body surface area; *LVM*, left ventricular mass; *LVMI*, left ventricular mass index; *RWT*, relative wall thickness; *FS*, fractional shortening; *LV S*, mean systolic velocity of the mitral annulus; *MAPSE*, mitral annulus plane systolic excursion; *LA*, left atrial dimension; *LA area*, left atrial area; *LA volume*, left atrial volume; *LAVI*, left atrial volume index; *LASR*, left atrial reservoir strain; *LASCD*, left atrial conduit strain; *LASCT*, left atrial contraction strain; *Ao*, aortic root; *E*, early mitral inflow velocity; *A*, late mitral inflow velocity; *E/A*, early-to-late mitral inflow velocity; *E’*, early diastolic mitral annular velocity; *A’*, late diastolic mitral annular velocity; *E/E’*, early mitral inflow velocity/early diastolic mitral annular velocity ratio

An absolute LV end-diastolic diameter, LA diameters, and the LV relative wall thickness (RWT) decreased significantly after surgery. However, indexation for body surface area (BSA) showed reversed associations, demonstrating the relative increase in the LV and LA size for BSA values after surgery.

**Fig. 2:**
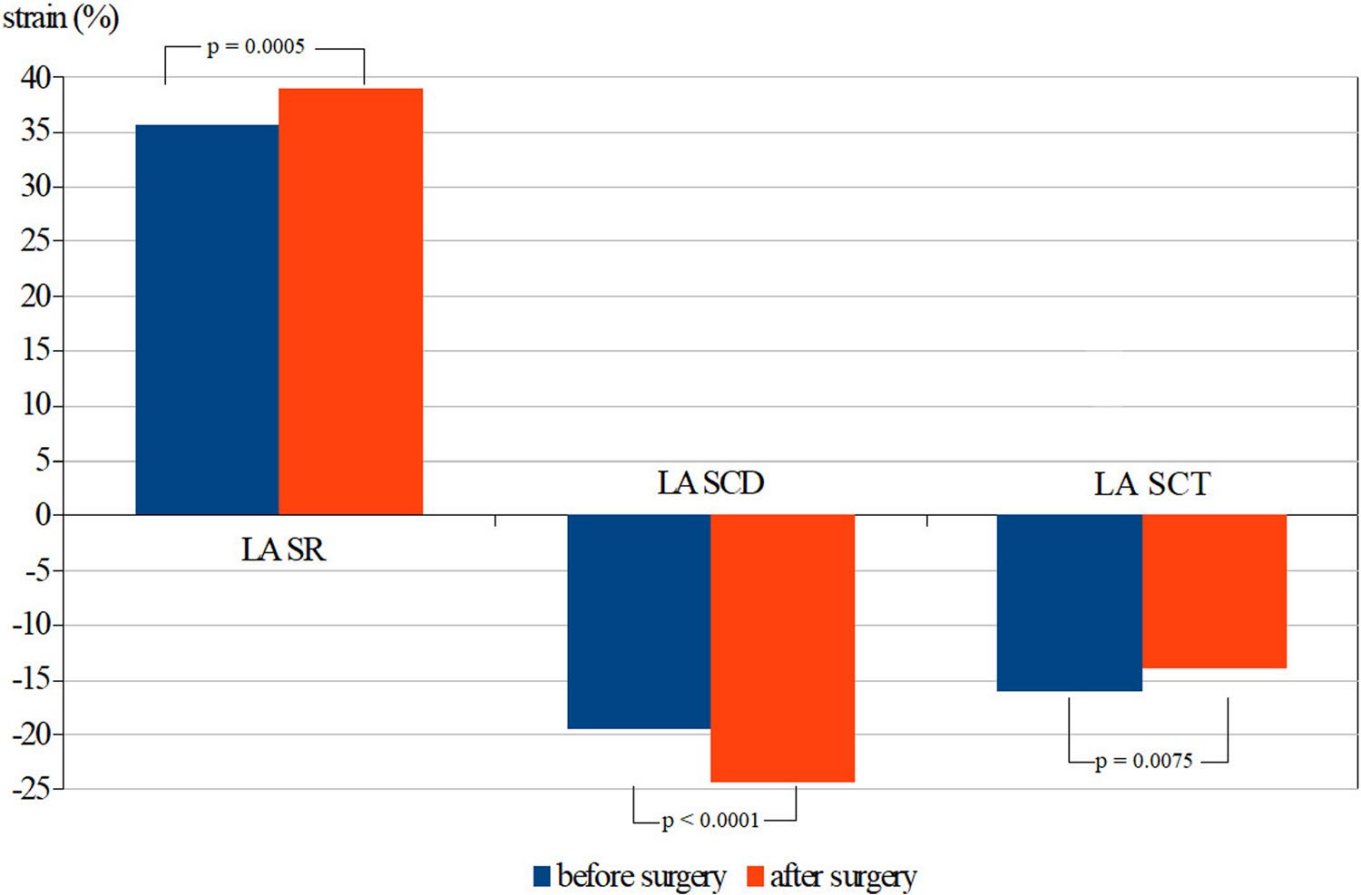
Changes in left atrial strain before and after bariatric surgery. LASR, left atrial reservoir strain; LASCD, left atrial conduit strain; LASCT, left atrial contraction strain

The spectral and tissue Doppler parameters of the LV diastolic function (*E*/*A*, *E*’, *E*/*E*’) significantly improved after surgery. Also, the systolic function parameters measured by mitral annulus systolic velocity, MAPSE, and FS increased significantly after surgery. There was a statistically significant improvement in all components of the LA strain measurements after surgical weight loss. LASR and LASCD significantly increased and LASCT significantly decreased (Fig. 2). The degree of increase in LASR correlated with the increase in *E*’ velocity and MAPSE (Spearman cc 0.38 and 0.32 respectively) and with the decrease in weight and *A*’ velocity (Spearman cc 0.27 and 0.27 respectively). The increase in LASCD correlated significantly with the increase in *E*’ velocity and MAPSE (Spearman cc 0.51 and 0.47 respectively). The decrease in LASCT correlated significantly with the decrease in *A*’ velocity (Spearman cc 0.27). There was no correlation between the change in any component of LA strain and the change in blood pressure, heart rate, left ventricular mass, or LA diameter.

The ICC for intraobserver repeatability of LASR was 0.98, and an absolute difference between 2 measurements/means of the 2 measurements was 4.71 ± 5.01%. The ICC for interobserver repeatability of LASR was 0.97, and an absolute difference between 2 measurements/means of the 2 measurements was 6.17 ± 4.31%.

## Discussion

The results of our study show, that in middle-aged individuals with severe obesity, and without known cardiac disease, surgically induced weight loss significantly affects all components of the LA strain, including increased LASR and LASCD and decreased LASCT. This beneficial influence of weight loss on the LA mechanical function may represent one of the mechanisms of the favorable effect of surgical weight reduction on the incidence of AF and HFpEF [25]. Our results also showed that obesity is one of the extra-cardiac conditions impairing atrial function, even in patients without concomitant AF or ventricular or valve disease. This LA abnormal function can lead to an abnormal pressure–volume relationship that affects atrial remodeling and cardiac performance, and can result in AF, heart failure (HF), and further major adverse cardiovascular events.

The LA strain provides an important insight into the cardiovascular pathophysiology in severely obese individuals. Among the parameters used, LASR describes elongation of the LA walls during ventricular systole, when the movement of the mitral annulus towards the apex with a closed mitral valve promotes atrial filling from the pulmonary veins. LASCD represents shortening of the left atrium during early diastole and reflects LA conduit function for the passage of the blood from the pulmonary veins into the left ventricle. LASCT characterizes further shortening of the left atrium during atrial contraction, which is responsible for adding extra blood volume to the left ventricle (about 15–30% of LV diastolic volume) at the end of diastole [26].

In our study, the increase in LASR and LASCD was significantly correlated with the improvement of the LV diastolic and longitudinal systolic function. This was previously reported in a large cohort of healthy subjects, demonstrating that LASR and LASCD describe not only intrinsic properties of LA walls, such as LA stiffness, but also additionally integrate information on the LV systolic and diastolic function [27]. In healthy subjects, LASCT seems to be the least dependent on LV function compared to other LA strain components [28].

An improvement in LV diastolic function was clearly demonstrated after bariatric surgery and it is believed to be one of the major factors responsible for the beneficial effect of weight loss on the reduced incidence of HF [29, 30]. An improvement in diastolic function may also contribute to the decreased risk of AF after weight loss by reducing LV diastolic pressure and therefore decreasing the hemodynamic load on the left atrium [31].

The association between worsening of the LV diastolic function and obesity is well known and described before in the pediatric populations [32, 33]. However, the mechanism of this association is not well understood. Both metabolic (e.g., insulin resistance, hyperglycemia, abnormal myocyte fatty acids accumulation and metabolism, inflammation), hemodynamic (increased blood pressure, heart rate, and cardiac output), and mechanical factors (e.g., pericardial constrain by epicardial fat accumulation) may contribute to this association [34, 35].

However, the influence of weight loss on the LV systolic function is even less clear. In patients with depressed systolic function, bariatric surgery led to an improvement in LV ejection fraction, but not in patients with initially preserved LV systolic function [36]. The meta-analysis of 23 studies demonstrated no significant effect of surgical weight loss on the LV ejection fraction [37]. However, studies incorporating echocardiographic two-dimensional speckle tracking LV strain analysis demonstrated improvement in LV longitudinal function after sleeve gastrectomy [38–40]. In our study, the analysis of the LV longitudinal strain, as well as LV ejection fraction by Simpson method, was not performed due to suboptimal visualization of the LV endocardium in most of the patients before surgery. Therefore, for the assessment of LV longitudinal function, we used the systolic velocity of mitral annulus by tissue Doppler imaging and MAPSE by M-Mode, which measures the longitudinal systolic displacement of the mitral annulus towards the apex. These measures indicated significant improvement of the longitudinal LV systolic function after bariatric surgery in our subjects. However, it should be noted that the LV longitudinal systolic function is closely related to the LV early diastolic relaxation. The latter depends in part on the recoil phenomenon, which provides in early diastole additional energy stored in systole in coiled myocardial collagen fibers [41].

In our group of patients, the increase in LASR and LASCD was paralleled by the decrease in LASCT, which was associated with the decreased mitral annulus late diastolic velocity *A*’, the tissue Doppler marker of the LA function. This suggests that an improvement of early LV diastolic function (as represented by LASCD and *E*’) leads to a relative decrease of atrial contribution to the LV diastolic filling and in consequence to the lower atrial mechanical work that may be protective against LA dilatation and dysfunction. In obesity, decreased early ventricular diastolic function and, in consequence, increased LASCT was described before [42]. It may represent the state of chronic left atrial pressure overload due to the increased LV diastolic pressure. Together with the obesity-related increase in heart rate and cardiac output, this may, over time, predispose to LA dilatation and LA wall fibrosis. However, it must be stressed that not only hemodynamic, but also obesity-related metabolic factors, like activation of the renin–angiotensin–aldosterone and sympathetic nervous systems, local and systemic inflammation, hyperinsulinemia, hyperleptinemia, and possibly lipotoxicity, may adversely affect the LA wall and therefore favoring the risk of atrial arrhythmia [43, 44].

Recent data demonstrated an increasing role of LA strain as a prognostic cardiac imaging biomarker in the prediction of HFpEF and AF [11–15, 45, 46], which surpasses in accuracy, the traditional LA measurements of the atrial size [11–15, 47]. Its promising prognostic role may result from the integration of combined information, not only about LA function alone, but also about the LV systolic and diastolic function. As weight loss leads to decreased risk of AF and HFpEF, one would expect that it leads also to the improvement in the left atrial functional parameters. This hypothesis was supported by our findings showing beneficial changes in LA strain after bariatric surgery. In the literature, we found no data on the influence of weight loss on the LA strain; therefore, we believe our novel findings could contribute additional data to better understand the complex associations between obesity and cardiac function.

## Limitations

The analysis of LA strain requires good quality echocardiographic images that may be difficult to obtain in a significant proportion of patients, especially those with the most severe obesity. In our cohort, it led to exclusion of 31% of the patients, who were initially evaluated for the LA strain analysis. Therefore, the echocardiographic LA strain analysis may not be applied in every patient with the most severe obesity.

With the retrospective character of the study, the referral bias for the follow-up echocardiographic examination cannot be excluded. However, the demographic characteristics of patients who attended the follow-up study (*n* = 93) were not significantly different from the initial cohort of 171 patients evaluated before surgery.

It must be noted that the interpretation of LASCT may be problematic similarly to the *A* wave velocity of the mitral inflow on the spectral Doppler, which is a well-known pitfall of a typical diastolic function assessment. The decrease in LASCT and *A* velocity may represent both improvement and worsening of the atrial function. In a young, healthy person and with improvement in LV relaxation the lower LASCT and *A* wave indicate lower atrial mechanical work due to lower atrial contribution to the LV diastolic filling. But, in severe diastolic dysfunction with increased left ventricular end-diastolic pressure, lower LASCT and *A* velocity indicate depressed LA function due to high atrial afterload. Moreover, shortly after termination of atrial arrhythmia, lower LA contraction strain and *A* velocity may indicate depressed function due to atrial stunning. However, in our group of relatively young patients without significant diastolic dysfunction (normal diastolic function or grade 1 of diastolic dysfunction) and without arrhythmia, the decreased LASCT most probably reflected a beneficial change in LA afterload due to improved LV diastolic function.

During our data analysis, we performed a routine indexation to BSA, and we found that BSA indexation introduced a paradoxical increase in LA and LV dimensions after surgery. We think that this finding is of no clinical significance, considering the clinical observations of reduced incidence of atrial fibrillation and heart failure after surgically induced weight loss [20–24]. We believe that these findings reflect well-known difficulties in cardiac chamber indexation in severe obesity [48]. Although an indexation of LV and LA for BSA is recommended by guidelines, the main drawback of this approach is an overcorrection that may lead to falsely decreased LV and LA dimensions in severely obese subjects [49, 50].

## Conclusions

The left atrial mechanical function measured by strain improves after bariatric surgery as evidenced by an increase in LASR and LASCD and the decrease in LASCT. It can be partially explained by the beneficial effect of weight reduction on the left ventricular diastolic and longitudinal systolic function. Decreased LA mechanical work may contribute to favorable LA remodeling and therefore the lower risk of atrial arrhythmia and HF after surgically induced significant weight loss.
